# A Triple-Network Dynamic Connection Study in Alzheimer's Disease

**DOI:** 10.3389/fpsyt.2022.862958

**Published:** 2022-04-04

**Authors:** Xianglian Meng, Yue Wu, Yanfeng Liang, Dongdong Zhang, Zhe Xu, Xiong Yang, Li Meng

**Affiliations:** ^1^School of Computer Information and Engineering, Changzhou Institute of Technology, Changzhou, China; ^2^School of Basic Medical Sciences, Jiamusi University, Jiamusi, China; ^3^School of Physics, Engineering and Computer Science, University of Hertfordshire, Hatfield, United Kingdom

**Keywords:** Alzheimer's disease, large-scale brain networks, triple-network, functional connectivity, dynamic cross-network interaction

## Abstract

Alzheimer's disease (AD) was associated with abnormal organization and function of large-scale brain networks. We applied group independent component analysis (Group ICA) to construct the triple-network consisting of the saliency network (SN), the central executive network (CEN), and the default mode network (DMN) in 25 AD, 60 mild cognitive impairment (MCI) and 60 cognitively normal (CN) subjects. To explore the dynamic functional network connectivity (dFNC), we investigated dynamic time-varying triple-network interactions in subjects using Group ICA analysis based on k-means clustering (GDA-k-means). The mean of brain state-specific network interaction indices (meanNII) in the three groups (AD, MCI, CN) showed significant differences by ANOVA analysis. To verify the robustness of the findings, a support vector machine (SVM) was taken meanNII, gender and age as features to classify. This method obtained accuracy values of 95, 94, and 77% when classifying AD vs. CN, AD vs. MCI, and MCI vs. CN, respectively. In our work, the findings demonstrated that the dynamic characteristics of functional interactions of the triple-networks contributed to studying the underlying pathophysiology of AD. It provided strong evidence for dysregulation of brain dynamics of AD.

## Introduction

As a neurodegenerative disease, Alzheimer's disease (AD) has an irreversible pathology, a long course of disease, and a progressive aggravation (1). Although the relationship between mild cognitive impairment (MCI) and AD is still inconclusive, some researchers believe that MCI is a transition period between normal aging and AD (2). Many studies report that cognitive decline and preclinical stages of impairment in AD are primarily due to disruption of brain networks. The episodic memory and executive functions of AD appear to be abnormal, and the related functions are closely related to the normal functioning and integrity of the brain network. Most of the brain networks involved in AD research are: default mode network (DMN), central execution network (CEN), and salience network (SN). The changes of DMN, CEN, and SN may be related to the pathological changes of AD (3–6). For example, important areas of the DMN are closely related to memory, among which the medial temporal cortex and hippocampal memory are strongly correlated. Research has shown that DMN exhibits functional connectivity disruption in AD (6, 7). Dennis et al. found that functional connectivity and network integrity decline gradually during normal aging, but decline more rapidly in AD patients, with the greatest impact on DMN (8). The main component of the SN is the inferior frontal cortex, which is closely related to cognitive function and plays an important role in cognitive control (9). CEN is a brain network implicated in human cognitive control, and its frontal regions are implicated in episodic memory. The nodes of the CEN include the dorsolateral prefrontal cortex and the lateral posterior parietal cortex. The study of CEN is helpful for the monitoring of brain network in MCI patients (10).

Functional Magnetic Resonance Image (fMRI) is a non-invasive brain imaging technology with high spatiotemporal resolution and good repeatability. More and more applications in the field of brain science. Resting-state fMRI(rs-fMRI) is the data obtained when the subject lies in the magnetic resonance scanner without any stimulation and task processing. Compared with the task state, the research subject's coma, anesthesia and other states are also included. In the resting state, the brain also has inherent neural activity patterns and can perform specific functions. It is of great significance to use rs-fMRI to study the topology of the brain network. The advent of fMRI has brought about a growing number of methodological tools for studying cognitive function and dysfunction (11–14). Effectively separating meaningful neural signals from fMRI images and constructing brain functional network has important research significance for its earlier disease prediction. However, most studies mainly use static functional connectivity (sFC) from fMRI. Large-scale dynamic functional network connectivity (dFNC) provides more context-sensitive, dynamic, and direct view at higher network level. It distinguishes brain network dynamics between normal and diseased populations (5, 13, 15–18). The characteristics of dFNC data are that time is constantly changing, reflecting brain activity that changes over time, and different functional connectivity networks can be obtained at different moments, in sharp contrast to sFC. Menon proposed a triple network model consisting of the DMN, SN and (19). Many studies have demonstrated that the dynamic interactions among triple-network (DMN, SN, CEN) are critical for complex cognitive tasks. These contribute to early recognition of Alzheimer's disease (6, 12, 20–24).

Independent Component Analysis (ICA) is a method based on data analysis, without a priori assumptions, it can separate and extract the physiological noises such as head movement, heartbeat and breathing in the BOLD signal, while the brain activity-related components can be separated and extracted. Signal components can form a network of related functional brain areas, which is an increasingly widely used data analysis method in rs-fMRI research (25). Although the components extracted by the ICA method have good reproducibility and reliability, the traditional ICA method applied to individual fMRI data cannot extract the common characteristics among a group of subjects (26). The Group Independent Component Analysis (Group ICA) method is based on the traditional independent component analysis, through the fusion of independent components across individuals, to construct a group independent component, thus reflecting the overall characteristics of the brain operating mechanism of a group of subjects (27).

**Figure 1:**
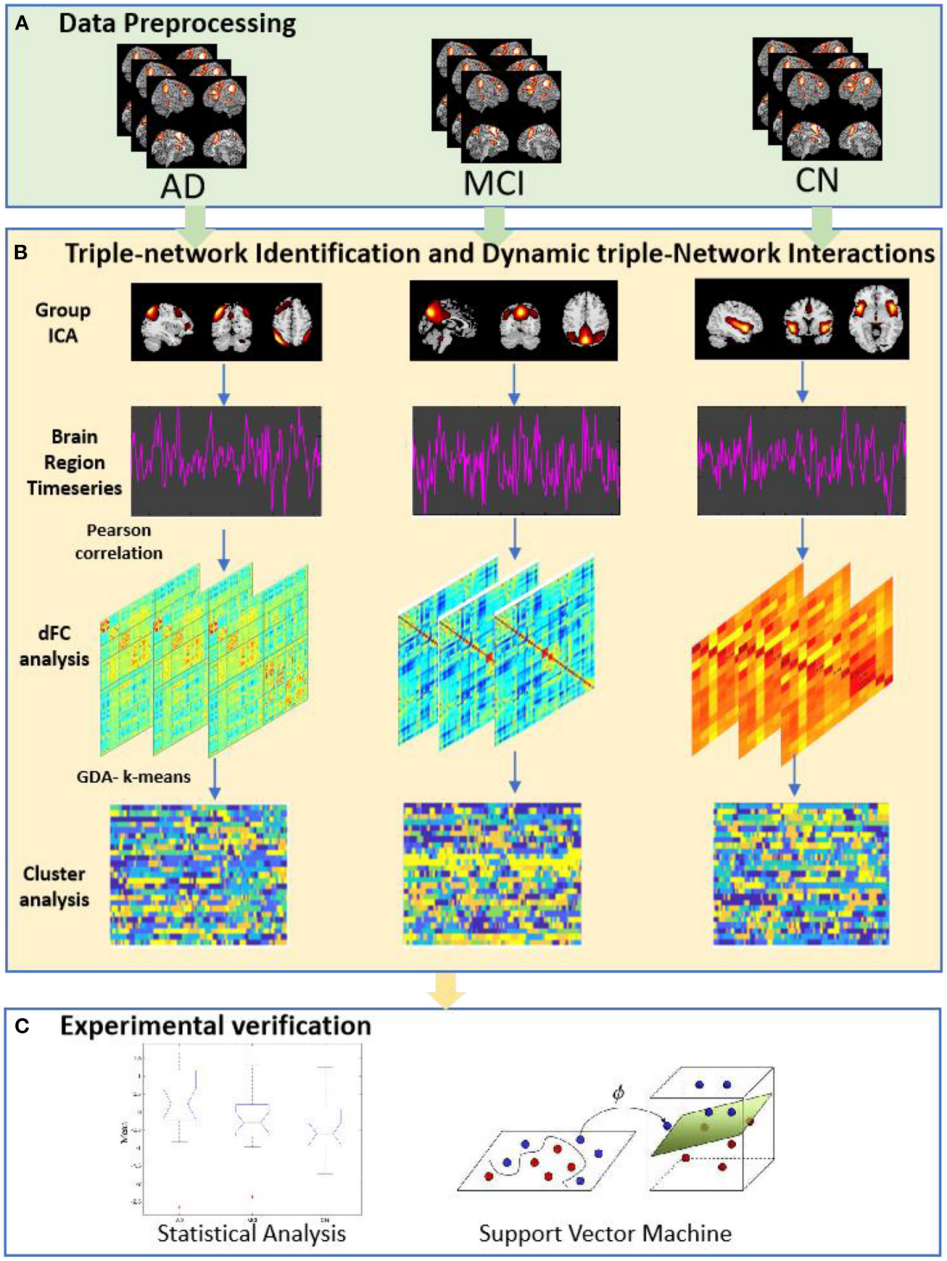
The study workflow. **(A)** Data Preprocessing, 145 rs-fMRI data were preprocessing. **(B)** Triple-network Identification and Dynamic Triple-Network Interactions. At this stage, group independent component was achieved; dynamic functional interactions were using a sliding window approach; cluster analysis based on k-means clustering method. **(C)** Experimental verification, were used statistical analysis and support vector machine approach.

In our work, we employed Group ICA to construct the triple-network (CEN, SN, and DMN) in AD, MCI and cognitively normal (CN). Then, we performed dFNC analysis of the three groups (MCI-AD-CN). Finally, we used statistical analysis and support vector machine (SVM) to validation. The study workflow was shown in Figure 1.

## Materials and Methods

### Subjects

The data for our study were obtained from the Alzheimer's Disease Neuroimaging Initiative (ADNI) database (www.adni-info.org). ADNI was established in 2003 at the initiative of Principal Investigator Michael W. Weiner, and was funded by the National Institutes of Health Security and the National Center for Aging Health and more than 20 private companies. It was designed to aid in the diagnosis of AD by using data including clinical diagnosis, neuroimaging, genetic material, and biomarkers. ADNI studies were conducted in ADNI-1, ADNI-GO, ADNI-2, and ADNI-3 phases. After three phases of research, the ADNI dataset has collected multiple multimodal data including MRI, PET, blood biomarkers, cerebrospinal fluid biomarkers (Aβ, tau protein) and genetic material. MRI was considered the preferred neuroimaging examination for AD. MRI data in the ADNI dataset were divided into three categories: AD, MCI, and CN subjects (28). fMRI was primarily measured by the relative levels of deoxyhemoglobin in each voxel, and rs-fMRI was to let the subject's brain completely empty, and to observe the characteristics of the interconnection of nerve fibers in the brain state without special activity (29).

In our study, rs-fMRI data (*N* = 145) included 60 cognitively normal (CN), 60 Mild Cognitive Impairment (MCI) and 25 AD subjects. The characteristics of the used subjects were shown in Table 1. The table showed that there were significant differences among the three groups in terms of gender (*p*<*0.001*), age (*p*<*0.001*), and MMSE (*p*<*0.001*).

**Table 1:** The clinical characteristics of subjects.

**Subjects**	**CN**	**MCI**	**AD**	***P***
Number	60	60	25	-
Gender (M/F)	17/43	29/31	14/11	<0.001
Age (mean ± sd)	72.7 ± 7.3	76 ± 8.5	76.7 ± 9.1	<0.001
MMSE (mean ± sd)	28.9 ± 1.3	27 ± 2.2	21.1 ± 3.9	<0.001

*CN, Cognitively Normal; MCI, Mild Cognitive Impairment; AD, Alzheimer's disease; MMSE, Mini mental status examination. T-tests were used for gender, age, and MMSE*.

### Data Preprocessing

MRI is one of the most commonly used methods for AD auxiliary diagnosis. MRI is divided into T1 and T2, where T1 is used to observe anatomical structures, T2 is used to display the lesion tissue. In this study, T1 is used as a reference image to which rs-fMRI data can be registered. The rs-fMRI (64 × 64 × 48) were obtained using an echo-planar imaging (EPI) sequence. Data preprocessing process: (1) Convert rs-fMRI from DICOM format to Nifti format using MRIcro software [MRIcro software guide (sc.edu)]. (2) We use SPM12 (30) and DPABI (31) for preprocessing as follows: (1) Remove the first 10 time points, Time points is set to 187, TR is set to 3 s; (2) Slice timing correction (slice number = 48, Reference slice = 48); (3) Realign and normalize: the MRI image of each subject is registered to the MNI standard space, and the volume distribution map of the brain gray matter is obtained after processing, and then the gray matter volume image of the registered standardized image is resampled to calculate 3 × 3 × 3 mm^3^ relative gray matter volume map (61 × 73 × 61); Normalize by DARTEL; (4) Smooth by using FWHM (full width at half maximum) of 8 × 8 × 8 mm^3^; (5) Band-pass filtering with 0.01~0.1 Hz; (6) Standardized settings: polynomial trend, frist on 24 head motion parameters, white matter signal, and cerebrospinal fluid signal.

### Triple-Network Identification

#### Group Independent Component Analysis

In our study, we employed group independent component analysis (Group ICA) to construct the triple-network (DMN, SN, and CEN). The principle of Group ICA was as follows: multiple subjects were compressed into fractions by principal component analysis (PCA) and then concatenated, the concatenated data was again reduced by PCA to reduce the fractions, and finally the mixed data was extracted by ICA to separate independent components. We set the ICA components to 30.

The mixed data of *M* subjects were reduced to the number of components by PCA, as shown in Formula 1.


(1) $$\begin{array}{l} {\bar{x}}^{\left(m\right)}={\left(F^{\left(m\right)}\right)}^{-1}x^{\left(m\right)},1\leq m\leq M\; \end{array}$$

Where ${\bar{x}}^{\left(m\right)}$ is the matrix of *L* × *V* after dimensionality reduction, *x*^(*m*)^ is the original mixture matrix of *T* × *V*, (*F*^(*m*))−1^ is the dimensionality reduction matrix of *L* × *T*, *T* and *V* are the number of time points and voxels of the data, and *L* is the time after dimensionality reduction dimension.

Then, we concatenated all the data after dimensionality reduction and used further dimensionality reduction as in Formula 2.


(2) $$X=Q^{\left(-1\right)}\times \left[\begin{array}{c} {\bar{x}}^{\left(1\right)}\\ {\bar{x}}^{\left(2\right)}\\ \vdots \\ {\bar{x}}^{\left(M\right)} \end{array}\right]=Q^{\left(-1\right)}\times \left[\begin{array}{c} {\left(F^{\left(1\right)}\right)}^{-1}x^{\left(1\right)}\\ {\left(F^{\left(2\right)}\right)}^{-1}x^{\left(2\right)}\\ \vdots \\ {\left(F^{\left(M\right)}\right)}^{-1}x^{\left(M\right)} \end{array}\right]$$

where *Q*^(−1)^ is the dimensionality reduction matrix of *N* × *LM*. After two PCA dimensionality reduction, the ICA algorithm was used to extract independent components. The ICA algorithm model was shown in Formula 3.


(3) $$\begin{array}{c} X=Â\hat{s}\; \end{array}$$

where Â is the mixture matrix of *N* × *N*, and ŝ is the matrix of *N* × *V* to represent *N* independent source signals.

Finally, it was reconstruction, using the estimated mixing matrix and source signal to reconstruct the time series and spatial images of *M* subjects, then the reconstructed source signal was as in Formula 4.


(4) $$\begin{array}{c} {\hat{S}}^{\left(m\right)}={\left({Â}^{\left(m\right)}\right)}^{-1}{\left(Q^{\left(m\right)}\right)}^{-1}\times {\left(F^{\left(m\right)}\right)}^{-1}x^{\left(m\right)} \end{array}$$

We employed GIFT toolkit (http://icatb.sourceforge.net/) for Group ICA (32, 33). We identified the DMN, SN, left CEN, and right CEN in 145 subjects.

#### The Analysis of DFNC

After extracting the BOLD time series of all ROIs of the subject, an exponentially decaying (ED) sliding-window strategy was applied to construct the dFNC of the brain. In this study, a sliding window of length *N* was selected, and the time series of length *L* was divided into *T* overlapping subsequences according to a certain step size *s*, where *T* = (*L* − *N*)/*s* + 1, then calculated the FNC matrix corresponding to each window.

The ED weights are computed as Formula 5.


(5) $$\begin{array}{l} w_{t}=w_{0}e^{\left(t-T\right)/\theta },t=1,...,T, \end{array}$$

where $w_{0}=\left(1-e^{-1/\theta }\right)/\left(1-e^{-T/\theta }\right)$, *t* is the *t*^*th*^ time point within the sliding window, *T* is the sliding window length (12), and the parameter θ controls the influence from distant time points. θ is set to a third of the window length (34). We set *T* to 39 s (TRs = 3 s) and sliding step to 1 s (35).

The definition of edge in brain network research was the functional connection between two brain regions. The dFNC between the two brain regions was determined by weighted Pearson correlation (wPC). It can reflect the interaction with time series of any two nodes *x*_*t*_ and *y*_*t*_ as Formula 6.


(6) $$\begin{array}{l} r_{w}=\frac{\sum_{t=1}^{T}w_{t}\left(x_{t}-\bar{x}\right)\left(y_{t}-\bar{y}\right)}{\sqrt{\sum_{t=1}^{T}w_{t}{\left(x_{t}-\bar{x}\right)}^{2}}\sqrt{\sum_{t=1}^{T}w_{t}{\left(y_{t}-\bar{y}\right)}^{2}}} \end{array}$$

where $\bar{x}=\frac{\sum_{t=1}^{T}w_{t}x_{t}}{T}$ and $\bar{y}=\frac{\sum_{t=1}^{T}w_{t}y_{t}}{T}$, then we calculated the z-transform of the weighted Pearson correlation.

### Dynamic Triple-Network Interactions

To make the clustering results independent of the number of subjects, 25 subjects with no significant differences in age and gender were selected from each group for cluster analysis. We explored group difference analysis based on k-means clustering method (GDA- k-means) (36).

The lower triangle (i.e., the six contiguous edges that do not repeat) of the weighted dynamic connectivity matrix *M* of 4^*^4 of each subject was extracted to form the data set *D*, *D* = {*m*_1_, *m*_2_, …, *m*_*x*_}. Afterwards, the functional connectivity matrix *M* of each group of subjects was clustered individually by using k-means. Since the distance function was not specified in the citation, the default Euclidean distance was used for the distance calculation, and the optimal number *K* of clusters was found using the sihouette maximum method (37), which finally *C* divided the sample set into *K* clusters. The process of clustering was as follows, firstly, k samples were randomly selected from the dataset *D* as the initial k prime vectors, which represented as μ = {μ_1_, μ_2_, …, μ_*k*_} *C* initialize the final cluster division as *C*_*t*_ = φ, (*t* = 1, 2, …, *k*). After that, the samples *x*_*t*_ and each center-of-mass vector were taken to μ_*j*_ calculate the Euclidean distance *d*_*ij*_, which was calculated as shown in Formula 7.


(7) $$\begin{array}{l} d_{ij}=\sqrt{{\left(m_{i}-\mu_{j}\right)}^{2}} \end{array}$$

The corresponding class corresponding to the *x*_*i*_ smallest *d*_*ij*_ fetch was noted as λ_*i*_ and the sample clusters were updated *C*_λ_*i*__ = *C*_λ_*i*__⋃{*x*_*i*_}. The above clustering steps were repeated and the sample clusters *C* = {*C*_1_, *C*_2_, …, *C*_*K*_} are finally output.

For each subject, we calculated the mean lifetime of each brain state based on the average time spent consecutively. We employed the brain state-specific network interaction index (NII) (9, 38) to characterize DMN, SN, and CEN network interactions (7).

The NII is calculated as shown in Formula 8.


(8) $$\begin{array}{l} NII=f\left(PC_{SN,CEN}\right)-f\left(PC_{SN,DMN}\right)\; \end{array}$$

where


(9) $$\begin{array}{l} f\left(PC\right)=\frac{1}{2}\ln\left(\frac{1+PC}{1-PC}\right)\; \end{array}$$

*PC* is Pearson's correlation between the time series of two networks, such as *PC*_*SN,DMN*_ refers to correlation between the time series of SN and DMN. *f* (*PC*) computes Fisher z-transform of Pearson Correlation (*PC*) between ROI time series. That is, the fisher-z changes of the functional connectivity coefficients of SN-LCEN and SN-RCEN were summed and subtracted from the fisher-z changes of the functional connectivity coefficients of SN-DMN.

### The Classification Based on Dynamic Triple-Network Interactions

To validation the robustness of differences in dynamic triple-network interactions, we calculated the mean of NII (meanNII) between the three groups. We took meanNII, age, gender as features and used a liner support vector machine (SVM) for classification (39). Due to the limitation of sample size, we applied the leave-one-out cross-validation method to evaluate the performance of the classifier, in order to ensure the reliability and stability of the results. The classification performance was measured by the classification accuracy, precision, recall, and specificity.

## Results

### Triple-Network in Alzheimer' Disease

**Figure 2:**
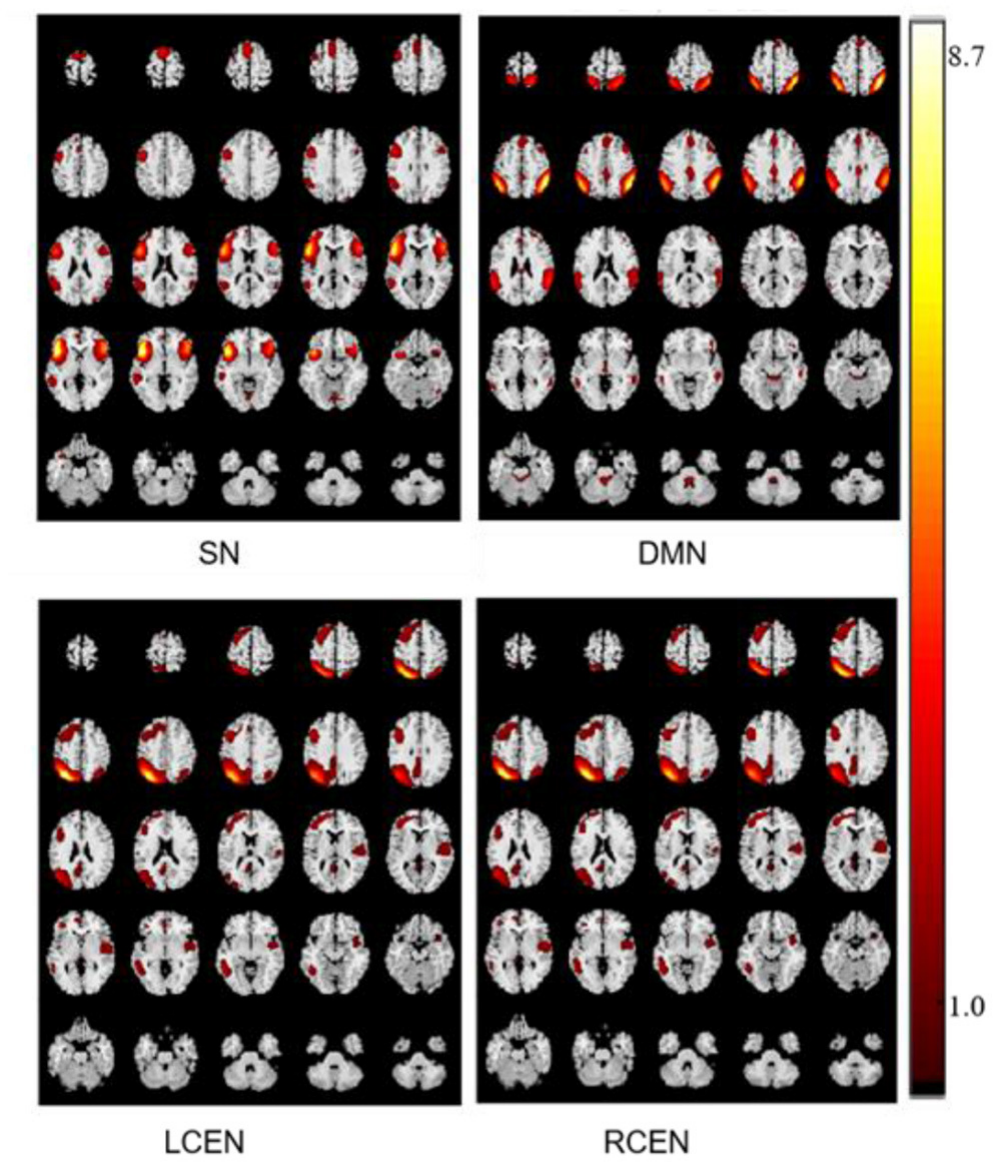
The spatial distribution of components in SN, DMN, left CEN, and right CEN.

Using template multiple regression methods to screen components 18, 29, 25, and 12, we identified four networks SN, DMN, left CEN, and right CEN in Alzheimer' disease. The spatial distribution of components was shown in Figure 2.

**Figure 3:**
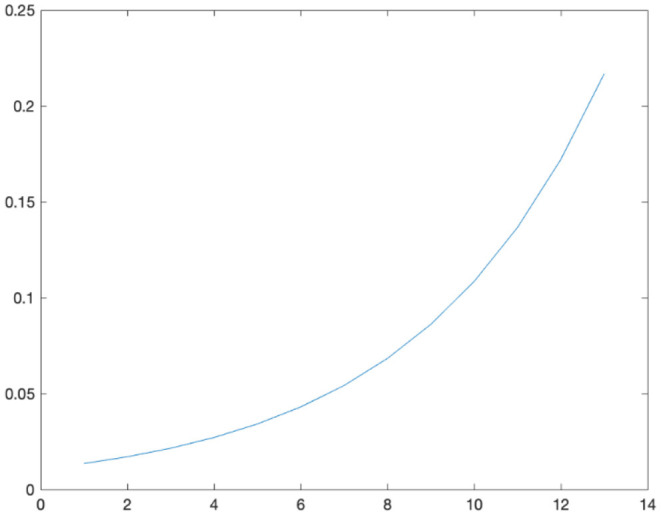
The variation of the window weight value.

Since the TR of data was 3 s, the window length was 39 s, that is, 13 time points, and the step size was 1 TR; the data was 187 time points, 175 time windows. The variation of the window weight value *w*_*t*_ with time *t* was as Figure 3.

**Figure 4:**
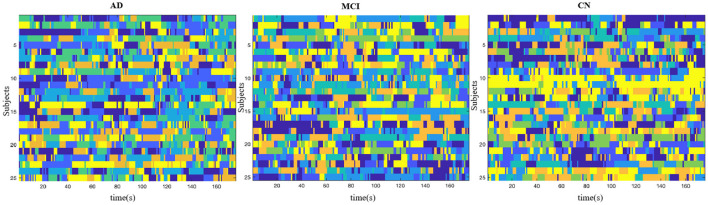
Brain States. AD group showed six states, MCI group showed seven states, and the CN group showed seven states. Color denotes distinct states in each subject.

We examined dynamic cs brain networks identified by the Group ICA and found six states in AD group, seven states in MCI group, and seven states in CN group. Brain States (BS) were shown in Figure 4.

### Dynamic Triple-Network Interactions

**Figure 5:**
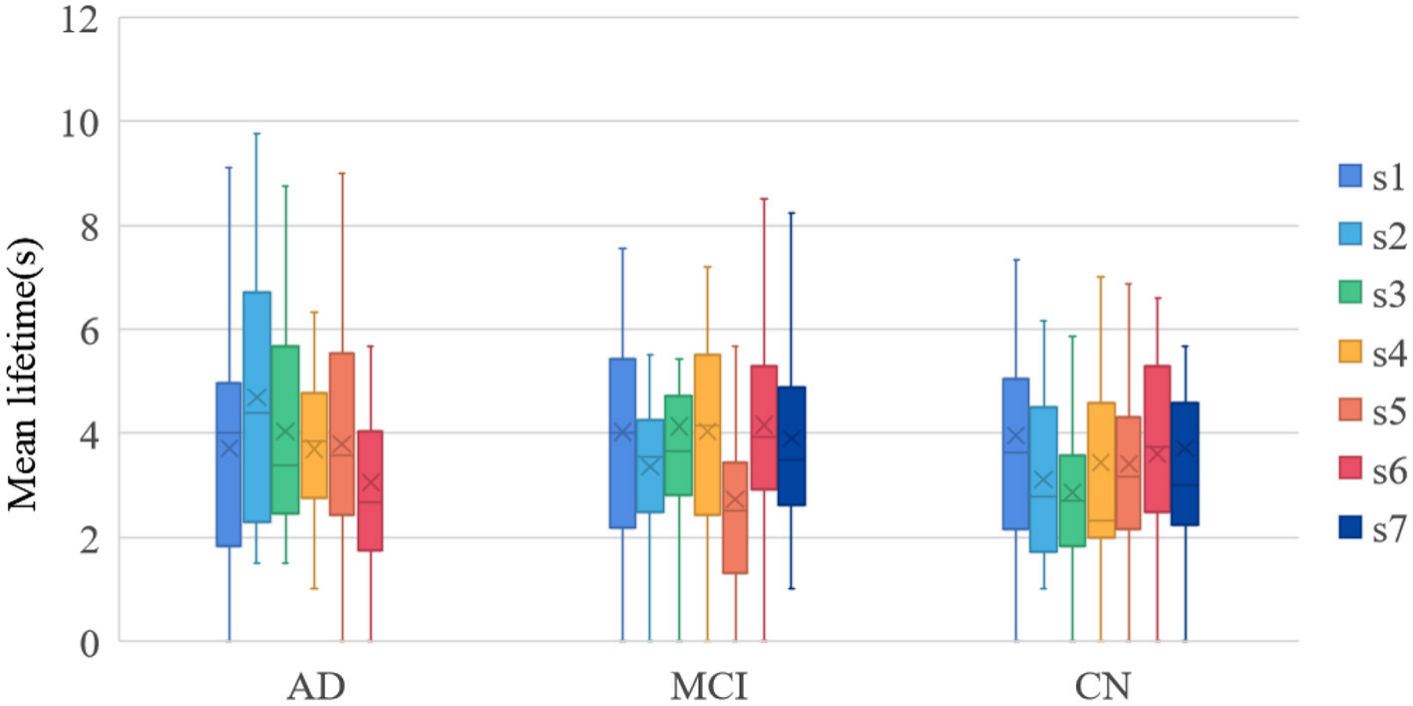
Mean lifetimes of dBS. s1~s7 were state 1~state 7.

In this study, the mean lifetimes of dynamic Brain States (dBS) were compared of the three groups. Mean lifetimes of dBS were shown in Figure 5. From the figure, the mean lifetimes of s2, s3, and s5 in CN group were more significant than in MCI group, but the mean lifetimes of s2 and s5 in AD group were significant than in the MCI group and CN group.

**Figure 6:**
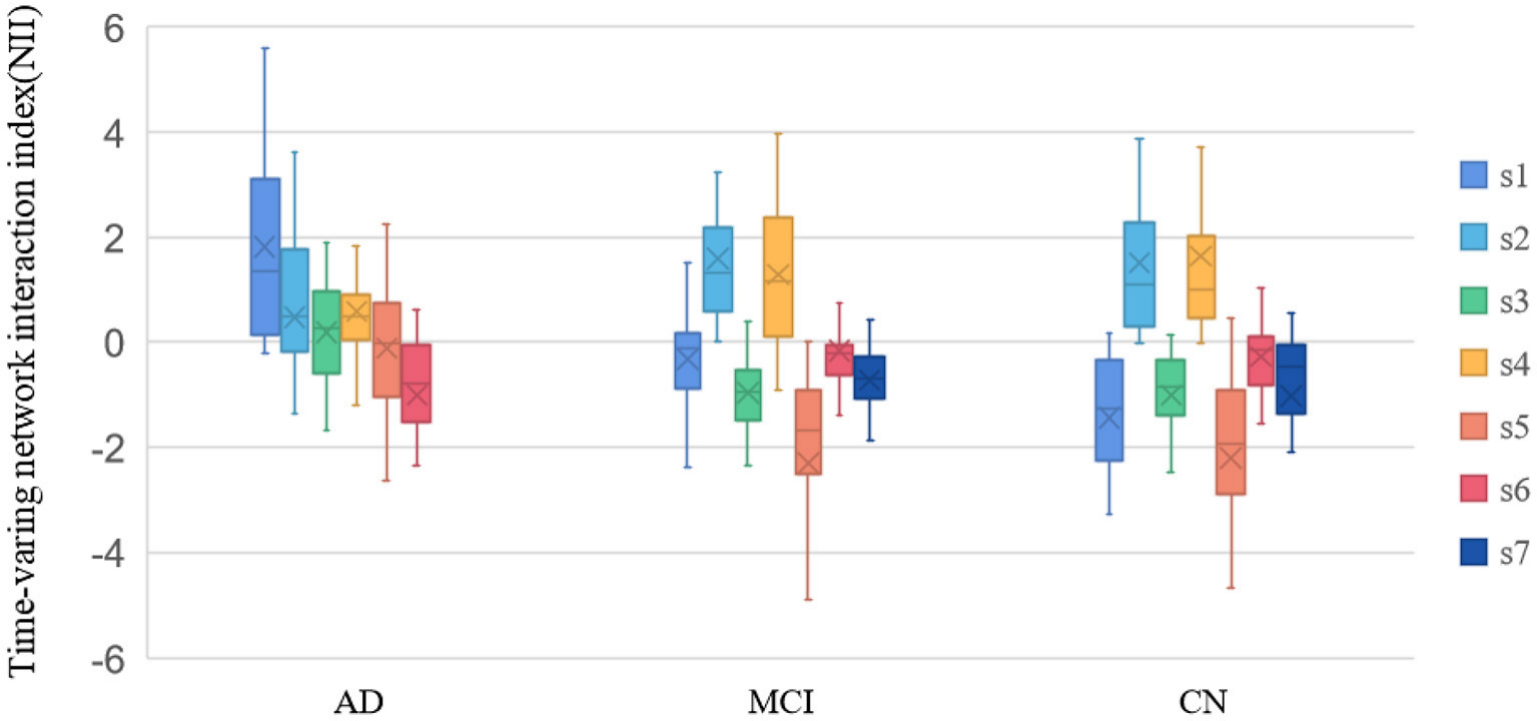
Time-varing network interaction index (NII). s1~s7 were state 1~state 7.

The meanNII in dBS was compared of the three groups. As can be seen from Figure 6, the meanNII of the s1, s3, s5 in AD group was more significant than in other groups (*p* < 0.05); the meanNII of the s1, s4 in MCI group was more significant than in CN group (*p* < 0.05).

### The Classification Based on Dynamic Triple-Network Interactions in AD

With the meanNII, age and gender as features, SVM was trained in three groups of AD, MCI, and CN. The results of the SVM for the classification were shown in Table 2. The method achieved 95% accuracy for distinguishing AD from CN, 94% for AD converters against CN, and 77% for CN converters against MCI.

**Table 2:** Predictive performance of the SVM classifier.

	**Accuracy**	**Precision**	**Recall**	**Specificity**
AD-CN	0.95	0.93	0.96	0.94
AD-MCI	0.94	0.96	0.91	0.93
CN-MCI	0.77	0.76	0.77	0.76

## Discussion

In our work, we aimed to explore the effective dynamic connectivity based a triple-network model in Alzheimer's disease. We identified three large-scale networks (DMN, SN, CEN) important for cognitive control and goal directed behavior in AD (i.e., from CN to MCI to AD) (7, 40). Due to the complexity and unobservable characteristics of brain networks, an indirect method should to be found to explore the network properties of the brain, and the group ICA method provided a feasible means for the study of brain networks. Group ICA estimates the activation area and time series of each network, and the time series of the network corresponding to the activation area may reflect the dynamics of the network.

As hypothesized, abnormal in AD patients through dynamic functional interactions. Dynamic triple-network coupling measures should predict Alzheimer's disease (41–43). We found that the dynamic functional network interactions of DMN, CEN, and SN was impaired in AD patients and that these abnormalities conduced to AD. Our findings were consistent with previous studies. Most of the information was directionally transmitted within the DMN, and the anterior default mode network was related to self-referential processing and emotion regulation, and the posterior default mode network was involved in consciousness and memory processing through its relationship with the hippocampus, which indicated plasticity. Information was constantly transformed in cognitive processes such as self-function, emotion, and conscious memory. The SN was involved in the detection and integration of cognitive and emotional information in the brain, indicating that as time progresses, cognitive and emotional information flowed into the SN for processing and integration. The CEN was involved in the regulation of cognitive control processing (19, 44, 45).

**Figure 7:**
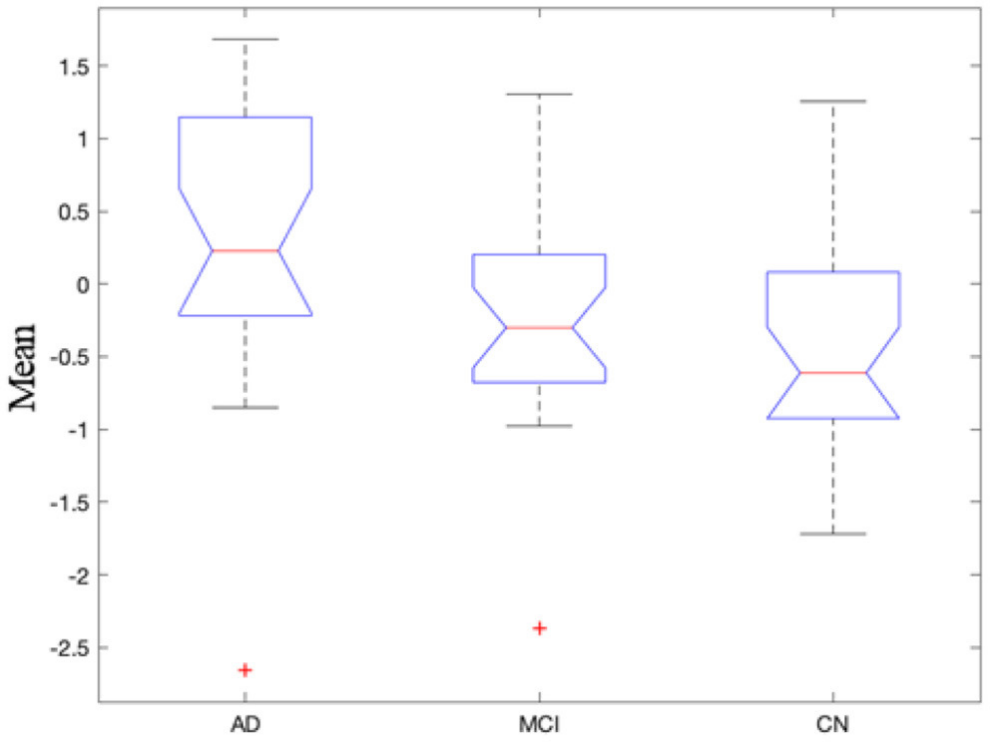
Mean of dynamic triple-network interactions in the three groups.

In recent years, there were a great deal of evidence of functional connectivity abnormalities inherent in Alzheimer's disease, but most studied static functional connectivity (46–50). However, there were few studies on dynamic functional connectivity and its relationship with clinical symptoms in Alzheimer's disease patients (51–53). The mean of NII in the three group (AD, MCI, CN) was analyzed by ANOVA, the difference was significant (*p* = 0.007). The NII-measured importance of dynamic triple-network interactions in the three groups were AD > MCI > CN (Figure 7). We computed the standard deviations of NII in the three groups (F = 1.87). The standard deviation of NII among the three groups was not significantly different (*p* = 0.16). The Pearson correlation analysis of mean NII and MMSE was calculated (r = 0.281, *p* = 0.087). Our findings demonstrated that the dynamic time-varying characteristics of functional interactions between triple-networks contributed to studying the underlying pathophysiology of Alzheimer's disease, as they captured the dynamic engagement of relevant brain circuits.

To verify the robustness of the findings, we implemented SVM method to examine the extent of differences among AD, MCI, and CN groups in relation to dynamic functional connectivity.

We found that dynamic triple-network interactions have high classification accuracy. This finding indicated that examining the features of dynamic triple-network states may illustrate positive relationships between DMN, SN, and CEN, contributed to understanding Alzheimer's disease.

In summary, our study demonstrated that large-scale functional network dysfunctions in Alzheimer's disease. In the future, studies should be needed to investigate the longitudinal stability of abnormal dynamics in different stages of Alzheimer's disease and to explore whether clinical outcomes differ from DMN, CEN, and SN dysfunction. Analysis of large-scale networks based on triple-networks has showed that they were powerful tools to study the core features of Alzheimer's disease.

## Data Availability Statement

Rs-fMRI data were downloaded from the Alzheimer's Disease Neuroimaging Initiative (ADNI) database (http://adni.loni.usc.edu/). Application for access to the ADNI data can be submitted by anyone at http://adni.loni.usc.edu/data-samples/access-data/. Further enquiries can be directed to the corresponding author.

## Ethics Statement

The ethical review was applied by ADNI. We applied and obtained the access from ADNI. The patients/participants provided their written informed consent to participate in this study. Written informed consent was obtained from the individual(s) for the publication of any potentially identifiable images or data included in this article.

## Author Contributions

XM, YW, YL, and LM led and supervised the research. XM, YW, and LM designed the research and wrote the article. XM and DZ performed data pre-processing. XM, YW, and ZX performed triple-network identification. XM, YW, YL, and XY performed independent component analysis, group difference analysis based on k-means clustering and SVM analysis. XM and LM did dynamic triple-network interaction analysis. All authors reviewed, commented, edited, and approved the manuscript.

## Funding

This study was funded by the National Natural Science Foundation of China (61901063), MOE (Ministry of Education in China) Project of Humanities and Social Sciences (19YJCZH120), Heilongjiang Provincial Natural Science Foundation of China (LH2021H103), Scientific Research Business Fund of Colleges and Universities in Heilongjiang Province (2019-KYYWF-1339), and the Science and Technology Plan Project of Changzhou (CE20205042, CJ20210155, CE20209003). This work was also sponsored by Qing Lan Project of Jiangsu Province. Data collection and sharing for this project was funded by the Alzheimer's Disease Neuroimaging Initiative (ADNI).

## Conflict of Interest

The authors declare that the research was conducted in the absence of any commercial or financial relationships that could be construed as a potential conflict of interest.

## Publisher's Note

All claims expressed in this article are solely those of the authors and do not necessarily represent those of their affiliated organizations, or those of the publisher, the editors and the reviewers. Any product that may be evaluated in this article, or claim that may be made by its manufacturer, is not guaranteed or endorsed by the publisher.
